# Origin of Magnetism in γ-FeSi_2_/Si(111) Nanostructures

**DOI:** 10.3390/nano11040849

**Published:** 2021-03-26

**Authors:** Liwei D. Geng, Sahil Dhoka, Ilan Goldfarb, Ranjit Pati, Yongmei M. Jin

**Affiliations:** 1 Department of Materials Science and Engineering, Michigan Technological University, Houghton, MI 49931, USA; ssdhoka@mtu.edu (S.D.); ymjin@mtu.edu (Y.M.J.); 2 Department of Materials Science and Engineering, Faculty of Engineering, Tel Aviv University, Ramat Aviv, Tel Aviv 6997801, Israel; ilango@tauex.tau.ac.il; 3 Department of Physics, Michigan Technological University, Houghton, MI 49931, USA; patir@mtu.edu

**Keywords:** origins of magnetism in nanostructures, density functional theory, density of states

## Abstract

Magnetism has recently been observed in nominally nonmagnetic iron disilicide in the form of epitaxial γ-FeSi_2_ nanostructures on Si(111) substrate. To explore the origin of the magnetism in γ-FeSi_2_/Si(111) nanostructures, we performed a systematic first-principles study based on density functional theory. Several possible factors, such as epitaxial strain, free surface, interface, and edge, were examined. The calculations show that among these factors, only the edge can lead to the magnetism in γ-FeSi_2_/Si(111) nanostructures. It is shown that magnetism exhibits a strong dependency on the local atomic structure of the edge. Furthermore, magnetism can be enhanced by creating multiple-step edges. In addition, the results also reveal that edge orientation can have a significant effect on magnetism. These findings, thus, provide insights into a strategy to tune the magnetic properties of γ-FeSi_2_/Si(111) nanostructures through controlling the structure, population, and orientation of the edges.

## 1. Introduction

Transition-metal silicides are important technological materials because of their advantageous properties, such as good electrical conductivity, thermal stability, and high chemical inertness [1,2,3,4]. Since Si is diamagnetic, a vast majority of bulk transition-metal silicides are nonmagnetic. For example, most bulk-size silicide crystals, based on the three typical transitional elements (Fe, Co, Ni), are reported to be nonmagnetic except for Fe-rich Fe_3_Si and Fe_5_Si_3_ [5,6,7,8]. One approach to inducing magnetism is fabricating epitaxial nanostructures. Epitaxial transition-metal silicide nanostructures often exhibit unique magnetic properties that are not present in their bulk constituents and, hence, have been the subject of intense research because of their technological potential in Si-based technology for spintronics devices [9,10,11,12].

In transition-metal silicide nanostructures, even Si-rich silicides can exhibit ferromagnetic ordering. For example, a ferromagnetic response has been observed for iron disilicide FeSi_2_ nanostructures, and such magnetic properties are not found in other transition metal silicides like NiSi_2_ or CoSi_2_ [13,14,15,16,17,18,19,20,21]. Multiple phases were reported for FeSi_2_ nanostructures, such as α, β, γ, and s. In particular, the CaF_2_-type γ-FeSi_2_, which is metastable in the bulk phase, can be stabilized in nanostructures with tunable magnetic properties [20,21,22,23,24]. Recently, the epitaxial self-ordering of γ-FeSi_2_ nanoislands on Si(111) substrate was fabricated, and magnetic properties were observed by measuring in-plane magnetization curves [20]. Most of such epitaxial Fe-silicide nanostructures exhibit apparent magnetic anisotropy, with clear in-plane orientation of the magnetization vector and only negligible opening of the hysteresis loop in the out-of-plane direction [25]. However, the origin of magnetism in γ-FeSi_2_/Si(111) nanostructures is still unclear. In order to explore the origin of the observed magnetism, we performed a systematic first-principles study.

In this study, we examined several factors that could contribute to the magnetism in nanostructures, such as the epitaxial strain, free surface, interface, and edge. The first-principles calculations show that only the edge can lead to magnetism in γ-FeSi_2_/Si(111) nanostructures, while the other factors merely suppress the magnetism. However, the magnitude of magnetism at different edges can be different, which exhibits a strong dependency on the local atomic structure of the edge. Furthermore, an enhancement of magnetism was observed in nanostructures with stepped facets that have multiple-step edges. Moreover, this study also reveals that the edge orientation can have a significant effect on the magnitude of magnetism. These findings suggest a potential strategy to tune the magnetic properties of γ-FeSi_2_/Si(111) nanostructures.

## 2. Methods

To study the magnetic properties of γ-FeSi_2_/Si(111) nanostructures, first-principles calculations were performed by using the Vienna Ab initio Simulation Package (VASP) [26,27,28,29]. These calculations are based on the periodic spin-polarized density functional theory (DFT). The plane-wave basis projector augmented-wave (PAW) method [30,31] was used in the generalized gradient approximation (GGA) with the Perdew–Burke–Ernzerhof (PBE) form of exchange–correlation functional [32]. To examine the effects of epitaxial strain, free surface, interface, and edge on the magnetic properties, we considered a unit cell of bulk γ-FeSi_2_, a nanoslab of pure γ-FeSi_2_, nanoslabs of γ-FeSi_2_/Si(111), with two different types of interface, and nanoislands of various epitaxial γ-FeSi_2_/Si(111) structures, respectively. For the supercells used to model slab and nanoisland systems, a vacuum space of over 15 Å was adopted to minimize the spurious interaction between the structure and its periodic images. We used the Monkhorst-Pack (MP) scheme with a 7 × 11 × 1 *k*-point grid to sample the Brillouin zone of the nanoslab systems, while a 1 × 11 × 1 *k*-point grid was used for the nanoisland systems. The cutoff energy of 500 eV was used for all our calculations. To optimize the slabs and nanoisland structures, the conjugate–gradient algorithm [33] was used to relax the ions into their instantaneous ground state. All atoms except bottom Si layers were allowed to relax until the residual force reached 0.01 eV/Å. Collinear spin-polarized calculations were performed to obtain the magnetic properties.

## 3. Results and Discussion

As mentioned before, to explore the origin of magnetism in epitaxial γ-FeSi_2_/Si(111) nanostructures, the contributions from several factors must be examined, including epitaxial strain, free surface, interface, and edge. Herein, we will investigate these factors systematically to determine the origin of magnetism unambiguously.

**Figure 1:**
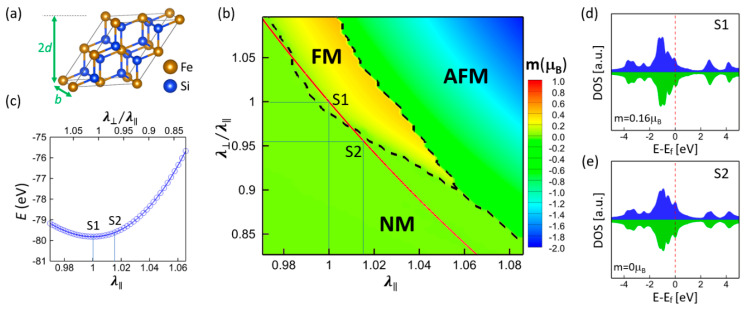
(**a**) γ-FeSi_2_ supercell with rhombohedral deformation of (111) in-plane stretch $\lambda_{∥}=\sqrt{2}b/a_{0}$ and out-of-plane stretch $\lambda_{⊥}=\sqrt{3}d/a_{0}$. (**b**) Magnetism mapping as a function of the rhombohedral deformation. The red line corresponds to the energy-minimized structure for a given $\lambda_{∥}$ and the color scale represents the calculated Fe spin moment m (positive for ferromagnetic (FM), negative for antiferromagnetic (AFM), and zero for nonmagnetic (NM)). (**c**) The calculated energy for the structures along the red line in (**b**). Magnetic moments and density of state (DOS) of Fe atoms for (**d**) S1 (unstrained state) and (**e**) S2 (epitaxially strained state).

Based on first-principles calculations, the lattice constant of bulk γ-FeSi_2_ (5.39 Å) was found to be slightly smaller than that of bulk Si (5.47 Å). Therefore, the Si substrate with (111) epitaxy is expected to exert a rhombohedral distortion on γ-FeSi_2_ nanostructures. In other words, such an epitaxial strain might be a possible factor that causes magnetism. To examine if it is epitaxial strain that leads to magnetic ordering, the relationship between magnetism and rhombohedral distortion (illustrated in Figure 1a) was calculated for the bulk γ-FeSi_2_. Figure 1b shows the mapping of magnetic states as a function of (111) in-plane stretch $\lambda_{∥}=\sqrt{2}b/a_{0}$ as well as out-of-plane stretch $\lambda_{⊥}=\sqrt{3}d/a_{0}$, where $a_{0}\;$= 5.39 Å is the lattice parameter of the unstrained bulk γ-FeSi_2_. The deformation can induce three magnetic states, namely, nonmagnetic (NM), ferromagnetic (FM), and antiferromagnetic (AFM). The in-plane strain $\lambda_{∥}$ is determined by the Si substrate, and the out-of-plane strain $\lambda_{⊥}$ (thus $\lambda_{⊥}/\lambda_{∥}$) that is along the film’s normal direction is free to vary. The corresponding optimized structure is depicted by the red line in Figure 1b, which reveals that only the phase transition between NM and FM states is induced by epitaxial strain. The calculated energy for the structures along the red line is shown in Figure 1c. Point S1 corresponds to the unstrained state ($\lambda_{⊥}=\lambda_{∥}=1$), and S2 corresponds to the strained state with the Si substrate epitaxial strain ($\lambda_{∥}=1.015$). It is noted that the unstrained bulk γ-FeSi_2_ exhibits an FM state, with a spin moment of 0.16 $\mu_{B}$ (S1), while the strained bulk γ-FeSi_2_ exhibits an NM state (S2). The corresponding density of state (DOS) of Fe atoms for S1 and S2 states are shown in Figure 1d,e, respectively. The epitaxial strain from the (111) Si substrate only changes the DOS profile of Fe atoms slightly. Rather than inducing the magnetic ordering, the epitaxial strain suppresses or even eliminates magnetism, indicating that epitaxial strain is not the cause of the observed magnetism.

**Figure 2:**
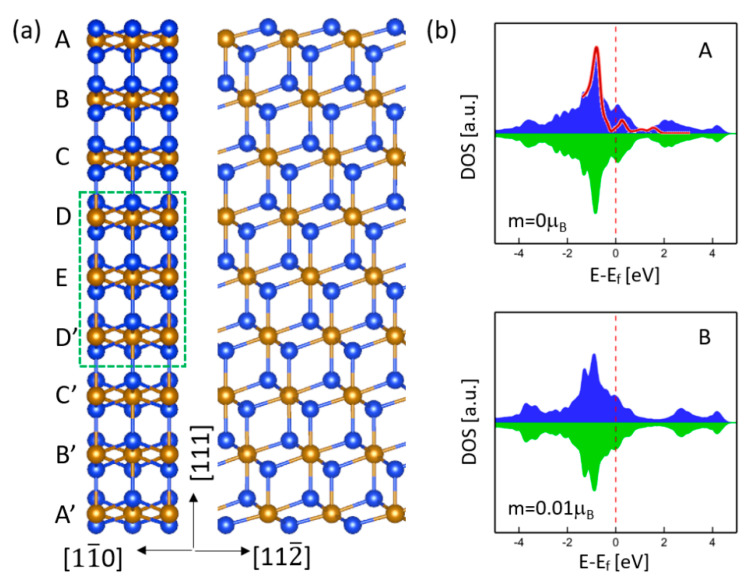
(**a**) Atomic structure of free-standing γ-FeSi_2_ (111) nanoslabs with nine Fe layers. (**b**) DOS of Fe atoms in the first two Fe layers (A and B) near the surface and the corresponding calculated spin moments. The red curve is an experimental DOS reproduced from [17].

To inspect the role of free surface on magnetism, a free-standing γ-FeSi_2_ (111) nano-slab with nine Fe layers was considered. Figure 2a shows the optimized atomic structure viewed from the $\left[1\bar{1}0\right]$ and $\left[11\bar{2}\right]$ directions. For this nanoslab, the epitaxial strain effect is excluded so that only the role of free surface on magnetism can be identified. The middle layers (D, E, D’) were fixed at the bulk lattice constant $a_{0}$ = 5.39 Å, which corresponds to Point S1 in Figure 1. Based on our calculations, the Fe atoms near the free surface exhibit negligible spin moments. For example, the spin moment of Fe atoms at Layer A is zero, while, at Layer B, it is nearly zero (0.01$\;\mu_{B}$). It can be expected that as the Fe layer is sufficiently far apart from the surface, the spin moment will approach the bulk value. Figure 2b shows the DOS of Fe atoms at the first two layers (A and B) near the surface, which is quite different from that of bulk γ-FeSi_2_ (Figure 1d). As expected, the DOS for the fixed middle layers manifests a similar DOS as the bulk shows. Looking into the DOS of first-layer Fe atoms more closely, we can find a sharp peak centered around −0.8 eV, which is in good agreement with the experimental measurements using in-situ scanning tunneling microscopy [17]. This sharp peak mainly originates from the Fe layer that is closest to the free surface. Our calculations reveal that starting from the second Fe layer, such a sharp peak begins to diminish. Thus, the presence of (111) free surface suppresses magnetism and results in a DOS that is distinct from that of the bulk. The same conclusion can be obtained even if considering the epitaxial strain effect. Our calculations confirm that when the middle three layers are fixed at the epitaxial film lattice constant (Point S2 in Figure 1), the film does not exhibit a magnetic ordering either.

**Figure 3:**
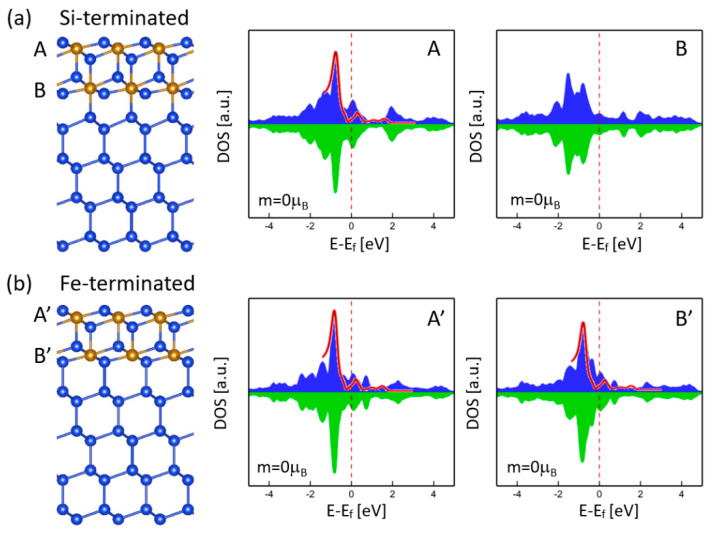
Atomic structures and DOS of Fe atoms in γ-FeSi_2_/Si(111) epitaxial nanoslabs with two types of interface: (**a**) Si-terminated and (**b**) Fe-terminated. The red curve is an experimental DOS reproduced from [17].

Unlike the epitaxial strain and free surface discussed above, the mismatch-induced atomic arrangements at the interface are expected to generate magnetism. For example, our previous calculations for γ-FeSi_2_/Si(001) systems show that the six-fold and seven-fold interfaces can exhibit a strong antiferromagnetic spin ordering, arising from the Fe(*d*)-Si(*p*)-Fe(*d*) superexchange interaction [34]. Since no atomic-level structural details of the interface are available *a priori*, to examine the interface effect on magnetism, γ-FeSi_2_/Si(111) epitaxial nanoslabs with two types of interface, namely, Si-terminated and Fe-terminated, were considered. For the Si-terminated interface, the Fe atom is eight-fold coordinated and, hence, saturated, while the Si atom has one unsaturated dangling bond, as shown in Figure 3a. For the Fe-terminated interface, the Fe atom is seven-fold coordinated and has one unsaturated dangling bond, while the Si atom maintains the tetrahedral coordination of bulk Si across the interface, as shown in Figure 3b. Both types of interfaces could be formed in the epitaxial self-ordering of γ-FeSi_2_ nanoislands, as indicated by the observations in other transition-metal disilicide nanostructures like CoSi_2_ [35]. Our calculations reveal that no magnetism is found for the two types of interfaces. The DOS spectra profiles of Fe atoms for Si-terminated and Fe-terminated interfaces are shown in Figure 3a,b, respectively. The Fe atoms at the free surface (A and A’) exhibit similar DOS as in the free-standing nanoslab without Si substrate (Figure 2b), all of which show the characteristic sharp peak centered around −0.8 eV that matches the experimental measurement [17]. On the contrary, the interface Fe atoms (B and B’) manifest different DOS features for both types. The characteristic peak disappears for the Si-terminated interface but remains intact for the Fe-terminated interface, probably because Fe atoms are in seven-fold coordination for both the free surface and the Fe-terminated interface. Although the two types of interface feature different DOS, neither of them contributes to magnetism.

**Figure 4:**
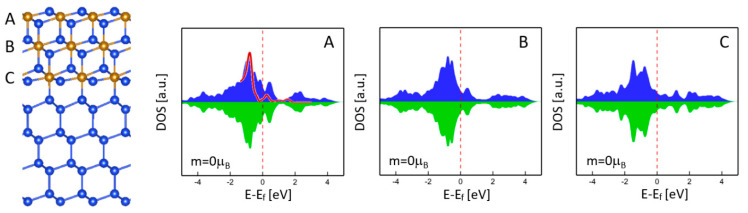
Atomic structure and DOS of Fe atoms in γ-FeSi_2_/Si(111) epitaxial nanoslabs with three Fe layers (**A**–**C**). The red curve is an experimental DOS reproduced from [17].

As mentioned above, the (111) free surface did not show any magnetism that could diminish or suppress the magnetism of nearby Fe layers due to the magnetic proximity effect. One may think that the magnetism of the interface might be eliminated by the NM free surface due to the short separation between the free surface and the interface, as shown in Figure 3. To understand this further, we considered the γ-FeSi_2_/Si(111) epitaxial nanoslab with three Fe layers (A, B, C), as shown in Figure 4. The DOS profiles of Fe atoms for the surface (A) and interface (C) were almost the same as in Figure 3a, but no magnetism could be found. Even for the nanoslab with more Fe layers, our calculations still did not show any magnetic ordering at the interface, which further confirms the nonmagnetic nature of the interface.

**Figure 5:**
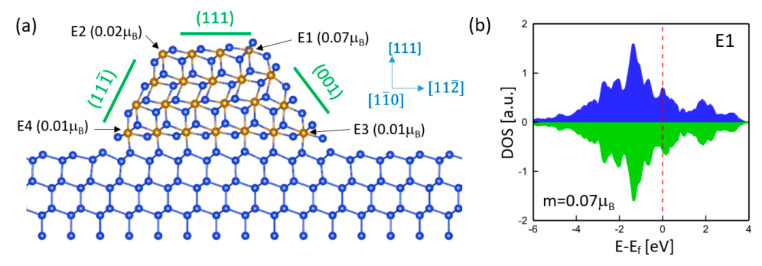
(**a**) Atomic structure and spin moments of γ-FeSi_2_/Si(111) epitaxial nanoislands with four Fe layers. The edges are denoted as E1, E2, E3, and E4, which are oriented along the $\left[1\bar{1}0\right]$ direction. (**b**) DOS of the Fe atom at edge E1.

Based on the above analysis, epitaxial strain, free surface, and interface do not contribute to magnetism. To examine the effect of edge, the γ-FeSi_2_/Si(111) epitaxial nanoisland with four Fe layers was considered, which include a (111) top free surface, a (111) bottom interface, a $\left(11\bar{1}\right)$ facet, and a (001) facet. Therefore, four edges along the $\left[1\bar{1}0\right]$ orientation, denoted as E1, E2, E3, and E4, are present in this nanoisland. The relaxed atomic structure is shown in Figure 5a. In fact, the epitaxial self-ordering of γ-FeSi_2_/Si(111) nanoislands prefers the <$1\bar{1}0$> orientations and most of the island edges and elongation directions are parallel to the <$1\bar{1}0$> orientations, as indicated by experimental observations of the epitaxial γ-FeSi_2_/Si(111) system [20]. Our computations show that Fe atoms at the surface, facets, interface, and interior exhibit zero spin moments and, hence, are NM, which is consistent with the above analysis. However, Fe atoms at the edges exhibit small but nonzero spin moments, indicating that the edge is the origin of magnetism in epitaxial γ-FeSi_2_/Si(111) nanostructures. Although edges can generate magnetic moment, the magnitude depends on the local atomic structure. For example, the Fe atom at surface edge E1 has a spin moment of 0.07$\;\mu_{B}$, while the other three edges (i.e., interface edges E3 and E4, as well as surface edge E2) only generate negligible spin moments. Figure 5b shows the DOS of the Fe atom at edge E1. The characteristic sharp peak at −0.8 eV disappears, which is different from that of free surface Fe atoms, as shown in Figure 2, Figure 3 and Figure 4.

**Figure 6:**
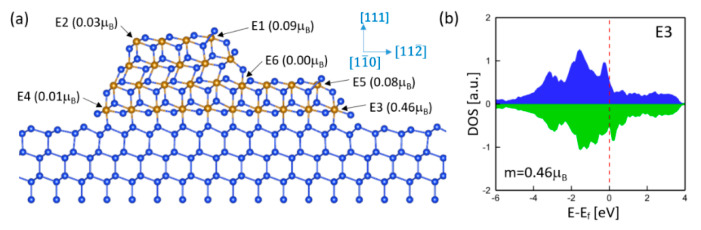
(**a**) Atomic structure and spin moments of γ-FeSi_2_/Si(111) epitaxial nanoislands with a step at the (001) facet. The edges are oriented along the $\left[1\bar{1}0\right]$ direction. (**b**) DOS of the Fe atom at edge E3.

Since edges can generate magnetism, the total magnetic moment is directly related to the population of edges. Large magnetism could be achieved by creating as many edges as possible. Usually, introducing steps can effectively enhance the edge population. Multiple edges and steps were observed for relatively thick γ-FeSi_2_/Si(111) epitaxial nanoislands [20]. To investigate the step effect, we considered the γ-FeSi_2_/Si(111) epitaxial nanoisland with a step at the (001) facet. The relaxed atomic structure, as well as the calculated spin moments, are shown in Figure 6a. It is noted that in comparison with Figure 5a, an additional edge is created by introducing a step at the (001) facet. Such a step edge is denoted as E5. As expected, almost all Fe atoms are NM except for those at the edges. In particular, the step edge Fe (E5) exhibits almost the same spin moment as the surface edge Fe (E1), which provides a major contribution to magnetism, indicating that introducing steps can effectively increase total magnetic moment in the nanoisland. Though the step creates another edge denoted as E6, E6 does not contribute to magnetism. It is worth noting that the step structure slightly enhances the value of spin moments of Fe atoms at edges E1 and E2 but significantly increases the spin moments at interface edge E3 when compared to the nanoisland without steps (Figure 5). The spin moment at E3 increases from 0.07$\;\mu_{B}$ to 0.46$\;\mu_{B}$, which is mainly attributed to its nearest Fe atom at step edge E5. The corresponding DOS of the Fe atom at E3 is shown in Figure 6b. Large asymmetry of majority and minority spin DOS is evident because of the large spin moment, which is quite different from that of interface Fe atoms, as shown in Figure 3 and Figure 4.

**Figure 7:**
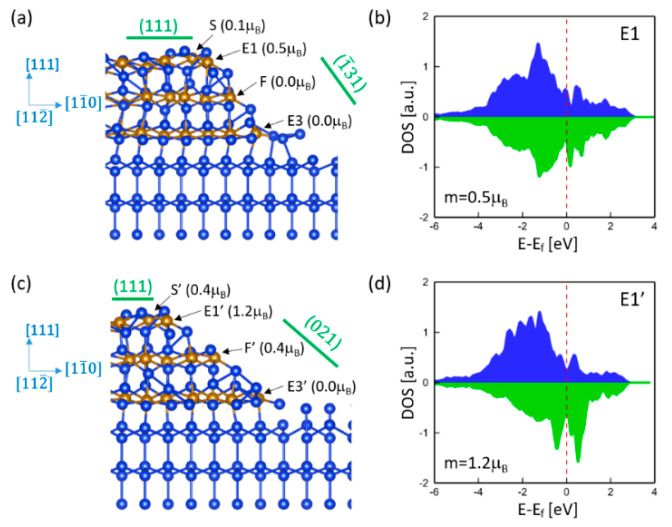
Atomic structure and spin moments of γ-FeSi_2_/Si(111) epitaxial nanoislands with (**a**) a $\left(\bar{1}31\right)$ facet and (**c**) a (021) facet. The edges are oriented along the $\left[11\bar{2}\right]$ direction. DOS of Fe atom at (**b**) edge E1 and (**d**) edge E1′.

Although the steps and edges of γ-FeSi_2_/Si(111) epitaxial nanoislands prefer to align along <110> orientations, some other possible crystallographic orientations could generate stronger magnetic moments. To investigate the orientation effect on magnetism, we considered γ-FeSi_2_/Si(111) epitaxial nanoislands with edges oriented along the $\left[11\bar{2}\right]$ direction. Two different facets were taken into account. Figure 7a shows the relaxed atomic structure of the nanoisland with the $\left(\bar{1}31\right)$ facet. It is noted that almost all Fe atoms are nonmagnetic except for the one at edge E1. The spin moment of Fe at E1 is 0.5$\;\mu_{B}$, which is much larger than that observed at the edge along the $\left[1\bar{1}0\right]$ direction. The Fe atom is six-fold coordinated and has two unsaturated dangling bonds that are responsible for a larger spin moment. Due to the magnetic proximity effect, such a large spin moment spills over to the nearest Fe atom (S) and results in a nonzero spin moment of 0.1$\;\mu_{B}$. Figure 7b shows the DOS of the Fe atom at E1, which exhibits a significant difference from that of the edge Fe along the $\left[1\bar{1}0\right]$ direction (Figure 5b) because of the distinct local atomic structures for the two edges. Figure 7c shows the relaxed atomic structure of the nanoisland with the (021) facet. The Fe atom at the interface edge (E3′) is still NM, but the Fe atom at the surface edge (E1′) exhibits a higher spin moment of 1.2${\;\mu }_{B}$. In fact, because of the smaller angle between the (021) facet and interface, it exhibits a step-like structure. For example, the Fe atom at F’ is six-fold coordinated and has a similar local atomic structure as that of edge E1′ and, thus, exhibits a much larger spin moment than the seven-fold Fe atom at F in the $\left(\bar{1}31\right)$ facet (Figure 7a). Thus, the step-like structure enhances the magnetism of nanoislands with edges oriented along the $\left[11\bar{2}\right]$ direction. Figure 7d shows the DOS of the Fe atom at E1′, which is more asymmetric than that of E1 (Figure 7b) due to the larger spin moment.

In this computational study, we only considered relatively small nanoislands that consist of three or four Fe layers, with only one step edge. In fact, the epitaxial self-ordering nanoislands are much thicker and wider, which depends on the initial Fe coverage. Based on the previous experimental work [20], 1~2 equivalent monolayers of initial Fe coverage (where 1 equivalent monolayer of Fe = 0.70 × 10^15^ atoms/cm^2^) can result in typical self-ordering nanoislands of 30~60 nm wide that consist of multiple edges. Most of these edges are oriented along the <$1\bar{1}$0> direction, while some of them are oriented along the <$11\bar{2}$> direction. Because of these multiple-step edges, a large magnetic response (with both large magnetization and large coercivity) has been observed via in-plane magnetization hysteresis measurement. However, higher coverage (for example, 2~10 equivalent monolayers of initial Fe coverage) can increase the probability of island formation in the midst of terraces, which leads to a reduced number of step edges and, thus, gives rise to a smaller magnetic response. This first-principles study agrees with the experimental results. Since the magnetization magnitude strongly depends on the population of edges, the total magnetism in epitaxial self-ordering γ-FeSi_2_/Si(111) nanoislands can be effectively enhanced by controlling the initial Fe coverage as well as the annealing temperature, which can also impact nanoisland geometry.

## 4. Conclusions

Magnetic properties were observed in epitaxial self-ordering γ-FeSi_2_ nanoislands on Si(111) substrate that are nominally nonmagnetic [20]. To explore the origin of the observed magnetism in γ-FeSi_2_/Si(111) nanostructures, we performed a systematic first-principles study. Regarding the specific geometry of the γ-FeSi_2_/Si(111) nanostructures, several factors could contribute to magnetism, including epitaxial strain, free surface, interface, and edge. To examine the magnetic properties contributed by these possible factors, we considered various nanostructure systems. The first-principles calculations show that epitaxial strain, free surface, and interface do not contribute to magnetism; only the edge causes magnetism and is, thus, responsible for the observed magnetism in γ-FeSi_2_/Si(111) nanostructures. The calculations show that the magnitude of magnetism strongly depends on the local atomic structure of the edge. Since only the edges contribute to magnetism, total magnetic moment can be effectively enhanced by creating as many edges as possible, which can be achieved by introducing steps during the fabrication process. This study also reveals that edge orientation can have a significant effect on the magnitude of magnetism. Our computational work is in good agreement with previous experimental observations [20], i.e., epitaxial γ-FeSi_2_/Si(111) nanoislands with more edges often exhibit a higher magnetization response. Thus, these findings provide insights into a strategy to tune the magnetic properties of γ-FeSi_2_/Si(111) nanostructures by controlling the structure, population, and orientation of the edges during the fabrication process.

## Data Availability

Data is contained within this article.
